# Emulsion Structural Remodeling in Milk and Its Gelling Products: A Review

**DOI:** 10.3390/gels10100671

**Published:** 2024-10-21

**Authors:** Dexing Yao, Le-Chang Sun, Ling-Jing Zhang, Yu-Lei Chen, Song Miao, Ming-Jie Cao, Duanquan Lin

**Affiliations:** 1 College of Ocean Food and Biological Engineering, Jimei University, Xiamen 361021, China; 2 Teagasc Food Research Centre, Moorepark, P61 C996 Fermoy, Co. Cork, Ireland; song.miao@teagasc.ie

**Keywords:** milk, emulsion structure, milk fat globule membrane, protein, nutrient

## Abstract

The fat covered by fat globule membrane is scattered in a water phase rich in lactose and milky protein, forming the original emulsion structure of milk. In order to develop low-fat milk products with good performance or dairy products with nutritional reinforcement, the original emulsion structure of milk can be restructured. According to the type of lipid and emulsion structure in milk, the remolded emulsion structure can be divided into three types: restructured single emulsion structure, mixed emulsion structure, and double emulsion structure. The restructured single emulsion structure refers to the introduction of another kind of lipid to skim milk, and the mixed emulsion structure refers to adding another type of oil or oil-in-water (O/W) emulsion to milk containing certain levels of milk fat, whose final emulsion structure is still O/W emulsion. In contrast, the double emulsion structure of milk is a more complicated structural remodeling method, which is usually performed by introducing W/O emulsion into skim milk (W_2_) to obtain milk containing (water-in-oil-in-water) W_1_/O/W_2_ emulsion structure in order to encapsulate more diverse nutrients. Causal statistical analysis was used in this review, based on previous studies on remodeling the emulsion structures in milk and its gelling products. In addition, some common processing technologies (including heat treatment, high-pressure treatment, homogenization, ultrasonic treatment, micro-fluidization, freezing and membrane emulsification) may also have a certain impact on the microstructure and properties of milk and its gelling products with four different emulsion structures. These processing technologies can change the size of the dispersed phase of milk, the composition and structure of the interfacial layer, and the composition and morphology of the aqueous phase substance, so as to regulate the shelf-life, stability, and sensory properties of the final milk products. This research on the restructuring of the emulsion structure of milk is not only a cutting-edge topic in the field of food science, but also a powerful driving force in promoting the transformation and upgrading of the dairy industry to achieve high-quality and multi-functional dairy products, in order to meet the diversified needs of consumers for health and taste.

## 1. Introduction

Milk is rich in macro- and micronutrients, including lipids, proteins, carbohydrates, vitamins and minerals [1]. These nutrients contribute to the growth and development of mammalian newborns. Among them, the lipids in milk and other dairy products are an important source of energy and nutrition for infants and even adults. For example, lipids in breast milk account for about 40–50% of an infant’s energy needs. Milk lipids are usually in the form of fat globules, which are natural colloidal particles that release energy-rich lipids (e.g., triglycerides) and various bioactive molecules (e.g., essential fatty acids, conjugated linoleic acid, phospholipids, sphingolipids, cholesterol, carotenoids, and fat-soluble vitamins A, D, E, and K). After infancy, humans continue to consume milk from non-human sources to supplement various nutrients, such as cow’s milk and ewe’s milk [2]. However, due to the potential microbiological risks of milk (e.g., the presence of pathogens, such as *Campylobacter*, *Salmonella*, and *E. coli*), as well as the instability of milk because of the large size of natural milk fat globules, commercially available milk often undergoes multiple processing operations to improve its safety, stability, and shelf-life, the most common of which is thermal treatment and homogenization [2].

Processing operations, such as homogenization or heating, can improve the stability and shelf-life of milk, and one of the most important reasons is their ability to alter the fat globule structure in raw milk and remodel the emulsion structure. In fact, milk, including cow’s milk, is itself an unstable oil-in-water (O/W) emulsion, in which milk fat globules (about 3.25 wt% in milk, ranging from 1 to 10 μm in diameter) encapsulated by a milk fat globule membrane (MFGM, containing highly polar lipids and proteins) are dispersed in ~90% water. Over time, this emulsion system may experience physical instability, such as changes in the arrangement or size distribution of milk fat globules, which can eventually lead to problems, such as flocculation, coalescence, and creaming [3]. However, after the processing of milk, its stability is mainly improved by reducing the size of emulsion droplets and reducing the number of microorganisms. The reduction in the size of the emulsion droplets leads to an increase in the interfacial area, but the newly formed interface is not completely covered by the MFGM, and casein can then be incorporated into the newly produced fat surface and thus provide stability against coalescence [1,2]. In addition, a lower microbial count reduces microbial spoilage and thus improves the safety of milk [4].

The structural remodeling of milk involves not only the modification of the particle size of the original milk fat globules, but also the change of the structure and configuration of the milk emulsion. Firstly, another oil/bubble can be used to replace the original milk fat globules to obtain milk with the structure of remolded single emulsion, which is generally based on skim milk for structural remodeling with the purpose of improving its taste and nutritional value. Second, a small amount of another O/W emulsion or a small amount of another oil can be mixed into the original O/W structure of the milk, such as fish oil [5,6], flaxseed oil [7], rapeseed oil [8] and sunflower oil [9], obtaining milk with a mixed emulsion structure, which is eventually still a kind of O/W emulsion. Finally, the original O/W structure of milk can also be transformed into water-in-oil-water (W_1_/O/W_2_) or oil-in-water-in-oil (O_1_/W/O_2_). For example, after removing the milk fat globules, another W/O emulsion system is introduced, and the W_1_/O/W_2_ composite/double emulsion structure is fabricated after mixing and homogenization. The purpose of this kind of milk emulsion structure remodeling is mainly to improve the physical properties of milk and construct milk with a specific functional property or suitable for a certain type of special population (e.g., consumers looking for functional foods and lactose intolerant patients), so as to produce new milk-based products and enhance their market competitiveness.

In recent years, there have been many in-depth studies on the structure of milk, but there is a lack of a systematic overview of the construction and application of milk with different emulsion structures and milk emulsion structure remodeling during processing. Therefore, the content of this review mainly focuses on the following aspects: (i) the original emulsion structure of milk and its remodeled single emulsion structure, (ii) the mixed emulsion structure of milk, (iii) the double emulsion structure of milk, and (iv) the changes of original/remodeled emulsion structures in milk during food processing, in order to provide researchers with a comprehensive understanding of the emulsion structure remodeling of milk.

## 2. Original Emulsion Structure and Restructured Single Emulsion Structure of Milk

**Figure 1:**
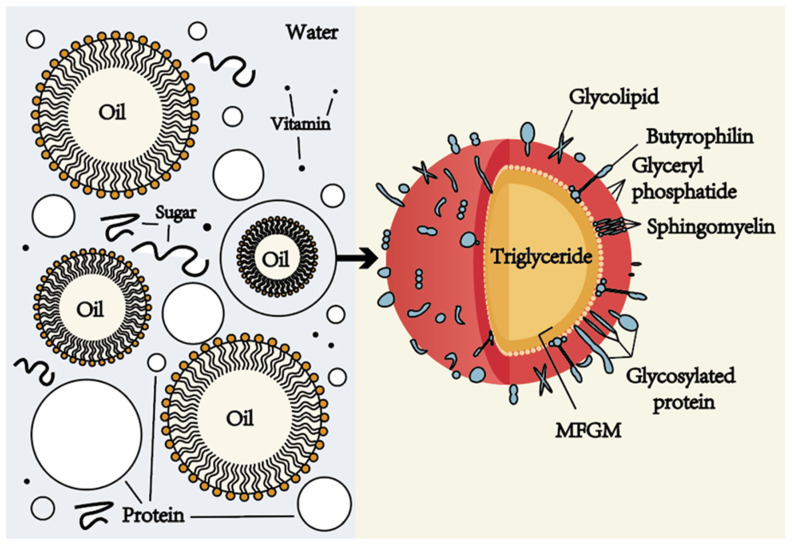
The original emulsion structure of milk. The left part of the figure shows the O/W emulsion structure of milk, in which the aqueous phase contains proteins, sugars, vitamins, etc., and the oil phase is composed of fat-soluble components, such as carotenoids, fat-soluble vitamins (A, D, E, K) and a variety of volatile flavor compounds. The right part is the structure of the milk fat globules, which are formed by a triglyceride core and coated by a milk fat globule membrane (MFGM) with a three-layer structure, where MFGM is composed of phospholipids, sphingolipids, cholesterol, glycoproteins and enzymes.

Milk is an emulsion composed of aqueous and oil phases, which is a chemically and physically unstable system. The aqueous and oil phases in an emulsion have different compositions and functions. The aqueous phase of milk is the continuous phase that is mainly composed of water, emulsifiers, and other water-soluble components, such as lactose and whey protein [10]. Water, as the main component of milk, mainly acts as a solvent in the milk emulsion, dissolving some water-soluble components, such as proteins, sugars, and vitamins, so that they are evenly dispersed in the milk emulsion system (Figure 1). At the same time, it also ensures the fluidity and taste of the milk. The oil phase of milk is the dispersed phase, which is usually composed of oil, emulsifiers, and other fat-soluble components, such as carotenoids, fat-soluble vitamins (A, D, E, K), and a variety of volatile flavor compounds [2] (Figure 1). The oil phase provides nutrients and taste, as the oils provide nutrients, such as energy and fat-soluble vitamins, while also imparting a rich mouthfeel and smoothness to the milk.

In milk, lipids account for about 3.5–5.2% of its total composition, mainly composed of triglycerides, accounting for more than 98% of the total lipids of milk, and the remaining, approximately 2%, of milk fats can be subdivided into different subcategories, mainly including diglycerides, monoglycerides, free fatty acids, phospholipids, and cholesterol [11]. The triglycerides in milk are in the form of MFGM-encapsulated spherical droplets, also known as milk fat globules. Structurally, milk fat globules are formed by a triglyceride-based core and surrounded by MFGMs with a three-layer structure, where MFGMs are composed of phospholipids, sphingolipids, cholesterol, glycoproteins, and enzymes [2] (Figure 1). MFGM plays a key role in the physical stability of triglycerides in milk because, as a natural emulsifier, it is able to form an interfacial adsorption layer on the surface of the lipid droplets, reducing the physical attraction between fat globules and reducing their tendency to aggregate, thereby reducing the flocculation and coalescence of fat globules [11,12,13]. In addition, MFGM can also protect fat globules from the action of lipase and slow down the decomposition of milk fat, because the MFGM membrane can act as a physical barrier to block the contact between lipase and fat globules, and MFGM also contains lipase inhibitors, which can slow down the action of lipase, thereby delaying the hydrolysis process of fat [2,11,12,14,15].

Although the presence of MFGMs in milk can reduce the surface tension between the aqueous and oil phases to a certain extent, the emulsified colloidal structure is still susceptible to interference and damage by external factors, which leads to the deterioration of the stability and quality of milk. There are two main triggers for this instability. On the one hand is the creaming caused by gravity, due to the different densities of oil droplets and water droplets, during which the oil may float to the top layer of the emulsion, leading to instability of the milk emulsion structure; on the other hand, the changes in temperature and pH can also affect the stability of milk. High-temperature treatment may melt emulsifiers with high melting points, losing the ability to stabilize the emulsion, and extreme acid-based conditions may alter the structure and properties of milk proteins, thereby disrupting the emulsion structure. In order to improve the stability of milk, some measures can be taken, such as adding appropriate emulsifiers, reasonably adjusting the pH and temperature of the emulsion system and adopting appropriate processing methods to prevent the occurrence of unstable phenomena, such as emulsion creaming [16,17,18].

**Figure 2:**
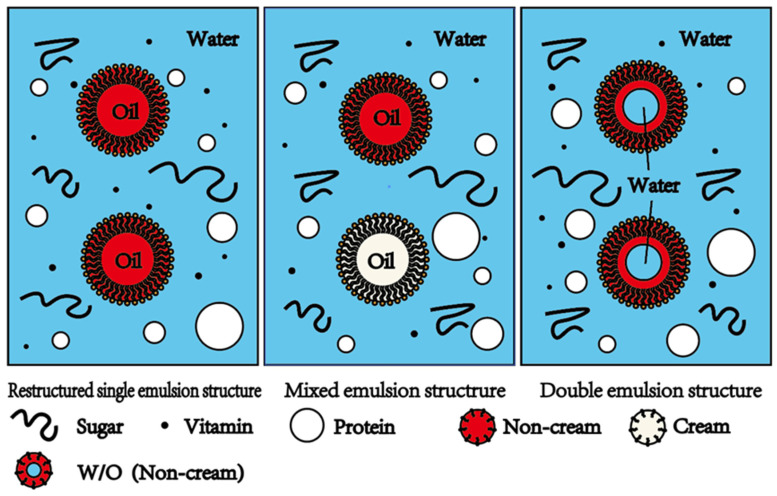
Milk with three different remodeled emulsion structures: restructured single emulsion structure, mixed emulsion structure, and double emulsion structure. Milk with the restructured single emulsion structure contains non-milk lipids. Milk with the mixed emulsion structure contains both milk fats and non-milk lipids. Milk with the double emulsion structure contains W/O emulsion droplets.

In addition, most of the fat in fresh milk can be removed by physical methods, such as high-speed centrifugation, and skim milk can be obtained. Skim milk is easy to store because it contains almost no fat, is less prone to oxidation, and is also beneficial for lowering cholesterol, blood pressure and triglycerides. However, skim milk does not taste as good as whole milk, and fat-soluble substances (vitamins A, D, E, K) are lost during the skimming process. Therefore, there are also a small number of studies on the structural remodeling of skim milk, mainly by introducing a small amount of another oil/bubble into skim milk to obtain milk with a restructured single emulsion structure, in order to improve the taste and nutritional value of skim milk (Figure 2). For example, the replacing of fat globules with bubbles in acidified milk matrices has been studied to develop better-tasting and low-fat products [19].

## 3. Mixed Emulsion Structure in Milk

Milk with mixed emulsion structure refers to the original structure of milk (i.e., the O/W emulsion structure) mixed with a small amount of another O/W or a small amount of another lipid substance (such as fish oil and flaxseed oil), and the final milk emulsion contains multiple lipids but it is still an O/W emulsion (Figure 2). The first step in fabricating the mixed emulsion structure is to add various admixtures to the original emulsion structure to obtain a mixed dispersion after mixing. These admixtures can be prepared with O/W emulsions and single or mixed oil phases (Table 1). Then, various processing methods are used, and the above mixed dispersion is emulsified to obtain a mixed emulsion. The aim of this kind of structural remodeling is to nurture the milk without affecting its stability, integrity and organoleptic properties, with the expectation of enhancing its beneficial effects on human health.

The addition of different admixtures may have different fortifying effects on milk. In terms of adding O/W emulsions, the nutritional value of milk has been enhanced by the addition of emulsions containing flaxseed oil or algae oil (Table 1), as they are both rich in omega-3 fatty acids. However, this method of structural remodeling may cause some adverse effects on milk. On the one hand, studies have shown that, with the increase of flaxseed oil addition (i.e., 8 wt%, 10 wt%, 12 wt%), the sensory score of milk decreases; therefore, an appropriate added concentration of oil should be selected and controlled to guarantee the nutritional fortification effect on milk without significant effects on its sensory scores [20]. On the other hand, the addition of oils rich in polyunsaturated fatty acids may reduce the oxidative stability of milk; therefore, it is necessary to add some antioxidants (such as ascorbic acid, ethylenediaminetetraacetic acid (EDTA) and sodium caseinate) to improve the oxidative stability of the mixed emulsion system [21].

In terms of adding lipids directly, many studies have been reported on remodeling the structure of milk by adding oils, such as fish oil, flaxseed oil, and sunflower oil. On the one hand, these enhance the nutritional value of milk. For example, fish oil and flaxseed oil are a class of oils rich in omega-3 polyunsaturated fatty acids (PUFAs) [6,7]; the addition of long-chain unsaturated lipids (e.g., canola oil) can balance lipid chain length and saturation levels to replicate the structure and nutrition of human milk [8]; the addition of sunflower oil to donkey milk lowers energy intake and improves its texture and health properties [9]; the introduction of lipids with specific biological activities, such as α-linolenic acid (ALA) and α-lipoic acid (LA), can also confer specific health effects on milk [23]. On the other hand, added lipids may also be beneficial in enhancing the physical stability of milk. For example, buttermilk powder is added to homogenized milk as an emulsifier for dairy products to produce milk emulsions containing a stable colloidal phase [15]; the addition of substances such as sweet buttermilk powder and cream residue powder, which are rich in natural emulsifier phospholipids, may also enhance the physical stability of milk [25,26].

Although, in most cases, the direct addition of lipids will reduce the oxidative stability of milk, this problem can be effectively solved by encapsulating the oil in advance (i.e., using spray drying to encapsulate the oil in food-grade wall materials, such as sodium caseinate, maltodextrin, and soy protein) or encapsulating it in liposomes to form nanoliposomes [6,23]. There are also studies of cow’s milk and soy milk supplemented with fish oil containing gallates, which have been shown to maintain the tocopherol content in the emulsion and inhibit lipid oxidation [22]. In addition, the direct addition of lipids with special flavors (such as fish oil) can adversely affect the flavor of milk. It has been found that the use of different homogenization temperatures and pressures to blend fish oil into commercial homogenized milk affected the fishy smell residue of fish oil [5]. The mixed milk emulsion after high-temperature and high-pressure homogenization (72 °C, 22.5 Mpa) is less fishy than that after low-temperature and low-pressure homogenization (50 °C, 5 Mpa); high-temperature and low-pressure (72 °C, 5 Mpa) emulsification also lead to a stronger fishy smell than high-temperature and high-pressure (72 °C, 22.5 Mpa) homogeneous emulsification, because the high temperature and high pressure can reduce the formation of volatiles [5].

## 4. Double Emulsion Structure in Milk

Liquid emulsion with a double emulsion structure refers to the transformation of the original O/W structure of milk into an O_1_/W/O_2_ or W_1_/O/W_2_ structure, which may involve multiple steps, including the removal of milk fat globules from milk and the introduction of another W/O emulsion system [16,27,28] (Figure 2). Table 2 shows the composition of the inner aqueous phase, oil phase, outer aqueous phase and emulsifier of milk with a double emulsion structure in several previous studies. Studies on introducing prepared W_1_/O/W_2_ emulsions into whole milk have also been reported [29]. However, such emulsion structures are complex, and precious research has mainly focused on skim milk with a double emulsion structure, which is therefore also the main focus of this review. unless otherwise specified.

Preparation of milk with a double emulsion structure enables fat replacement and the development of low-fat products. For example, W_1_/O/W_2_ milk has been prepared with skim milk, PGPR, and different alternative non-dairy fats, including LTVF, BF, HS, and SO, which may serve as a potential lipid-lowering alternative to full-fat dairy products [30]. However, this fat substitution may affect the subsequent processing performance of milk. For example, a study on how olive oil-based (W_1_/O/W_2_) composite milk emulsion affects curdling behavior indicated that the restructured milk had the lowest cheese yield compared to whole and low-fat milk [31]. Although olive oil-based restructured milk is not ideal in terms of cheese yield, this study provides a reference for the replacement of cheese milk fat with olive oil emulsions, and also provides a basis for the application of milk with a double emulsion structure in the development of other milk fat substitute products [31].

In addition, the double emulsion structure can also be fabricated for milk fortification. Some studies have used water-in-oil (W/O) emulsification to encapsulate iron, glycyrrhizic acid (GA) or vitamin B_12_ to prepare nutrient-fortified milk [17,18,32]. It is important to note that iron in milk may catalyze lipid oxidation, which subsequently leads to rancidity, producing unpleasant odors and tastes. Therefore, in order to prevent lipid oxidation in iron-micro-capsulated milk, the amount of added iron needs to be controlled within a small range (i.e., 0.1–0.3%, *w*/*v*) [32].

## 5. Remodeled Emulsion Structures in Gelled Dairy Products

Some studies have mainly focused on the effect of the original structures on the properties of gelled dairy products with the purpose of improving their taste and nutritive quality [33,34,35]. For example, it has been indicated that buffalo set-yoghurts made with unhomogenized milk exhibited higher syneresis and poor stability upon shear-induced breakdown, mainly due to the porous gel structure containing a large number of bigger fat globules [36]. Generally, the purpose of fabricating the emulsion structures of gelled dairy products is to use health-beneficial lipids, like those derived from plants, to replace saturated fatty acids [37]. Therefore, in most cases, remodeled emulsion structures in gelled dairy products include restructured single, mixed and double emulsion structures [31]. Milk can be processed along with other gelled/semi-gelled dairy products, including fermented (e.g., cheese and yogurt) and condensed milk products. Therefore, the first strategy in fabricating the emulsion structures in gelled dairy products is implying milk with pre-fabricated emulsion structures to produce dairy products with different emulsion structures [31]. For example, recombined milks were formulated using two different concentrations (4 g/100 g and 6 g/100 g) of spray-dried emulsions containing PUFA-rich oils encapsulated with buttermilk, followed by fermentation to produce yogurt [38]. However, milk with different fabricated emulsion structures can affect the processing and quality of its gelling products. It has been found that skim milk containing olive oil-based W_1_/O/W_2_ emulsions showed a shorter reticulation phase and a lower cheese-making yield than full-fat milk [31].

Another strategy is processing skim milk or whole-fat milk followed by directly fabricating the emulsion structures in gelled dairy products [39]. For instance, the replacement of milk fat by rapeseed oil stabilized emulsion with an oil content of 25%, 50% or 75% (*w*/*w* basis) in full-fat or free fat commercial yogurt has been investigated in a previous study [37]. The findings indicated that incorporating rapeseed oil stabilized emulsion with 75% oil would seemingly reduce oil droplet size without much compromise to bacterial viability, sensory, or texture, and that such a fat replacement strategy shows promise in dairy products [37].

## 6. Remodeling Emulsion Structures of Milk During Food Processing

Milk is highly nutritious, which also means that it is prone to spoilage and is an excellent substrate for the growth of disease-causing microorganisms. This poses a huge challenge for the dairy industry in providing safe, shelf-stable, and affordable milk [40]. As a result, various processing techniques are applied to extend the shelf-life, improve the taste and texture, enhance the stability, and improve the nutritional value and functionality of milk. The common processing methods include heat treatment [41,42], high-pressure processing [43,44], homogenization [34,45], ultrasound [10,46], micro-fluidization [35,47], freezing [48,49,50] and membrane emulsification [51,52,53]. These processing technologies are widely used in the manufacture of milk, dairy products, and dairy ingredients.

### 6.1. Thermal Treatment

Thermal treatment is one of the major traditional food processing methods. Heat treatment of milk reduces microbial spoilage by destroying the proteins and nucleic acids in the cells, cell wall and cell membrane structure of microorganisms, and thus provides safer products with an extended shelf-life [40]. Although intense heat treatment of milk components can improve the safety and shelf-life of milk, the flavor and nutritional value are negatively affected, accompanied by many other drawbacks, such as protein denaturation and modification, reduced nutritional value, and the formation of Maillard products. Heat treatment also affects mineral and vitamin balance, leading to undesirable changes in the flavor and color of milk [4,26,54].

Heat treatment may adversely affect the stability of the original emulsion structure and restructured single emulsion structure in milk for the following reasons. Firstly, coagulation in milk during thermal processing is one of the biggest challenges in dairy sterilization. Several reactions have been found to occur during heating, including denaturation of whey protein and the formation of complexes between denatured whey proteins, casein micelles, and milk fat globules, leading to the occurrence of thermocoagulation [12,26,55]. Therefore, in milk emulsion systems with high concentrations of proteins, such as (recombinant) evaporated milk, the thermal stability of proteins during heating, especially whey proteins, is very important for milk products [12,13]. The mechanism of thermal instability of dairy proteins is that heat treatment leads to the unfolding of the natural dense structure of globulin, which exposes buried hydrophobic residues and leads to the aggregation of denatured proteins [41]. Studies have shown that thermally induced protein instability and calcium phosphate thermo-precipitation are the main causes of scale on some parts during the thermal processing of milk. Under certain conditions, whey protein, casein, calcium phosphate and fat can be contained in the scale. When milk is heated, *κ*-casein may undergo heat-induced dissociation at temperatures greater than 60 °C, reducing the stability of casein micelles. In addition, pH (from 6.6 to 5.5) is a major factor in heat-induced *κ*-casein dissociation, which collapses casein micelles during acidification due to charge neutralization [40].

Second, heat treatment of milk can also affect lipid digestion. Studies have shown that heat treatment may alter the structure of the interfacial coating surrounding the fat globules, such as protein denaturation and cross-linking, making lipase molecules more difficult to adsorb. In addition, heat treatment may promote a greater degree of fat globule flocculation, which again limits the ability of lipase to adsorb on the surface of fat globules, thereby reducing the initial rate of lipid digestion [2]. Temperature also has a significant impact on the stability and integrity of MFGMs. Studies have shown that, in milk, heat treatment between 60 °C and 95 °C affects the surface structure of milk fat globules, mainly by whey protein binding to MFGM protein [11,56].

Third, during thermal processing, the degradation of lipids, proteins, and other emulsion components leads to change in the flavor profile of milk, which is associated with the formation of volatile compounds [12]. It has also been shown that heat treatment of milk also produces methyl ketones, such as 2-pentanone, 2-heptanone, and 2-nonanone, and 2-heptanone, which has been identified as one of the compounds with the strongest volatile flavor in hot milk [12].

Ultra-high-temperature heat-treated (UHT) milk has a long shelf-life at room temperature, making it a nutritionally, technically and economically important food. Studies have shown that certain chemical and physical changes caused by UHT treatment can lead to storage instability, including the formation of protein precipitates at the bottom of the storage container or the formation of precipitates or gels throughout the milk (mainly involving the aggregation of milk proteins), which limits the shelf-life and market potential of UHT milk [42]. However, the heat load after UHT (including evaporation and spray drying) generally has little effect on the physicochemical and functional properties of the production of infant formula (IMF) powders [57].

Indeed, consumer acceptance of milk is largely determined by its organoleptic characteristics and nutritional value, so there is a huge demand for new technologies to replace conventional thermal processing with technologies that cause minimal damage to nutrients and provide a longer shelf-life. To achieve this, a number of non-thermal techniques have been explored, such as high-pressure processing and ultrasonics [4,54].

There are currently no studies on the effect of heat treatment on the structure of milk with mixed or double emulsion structures. Heat treatment may affect the stability, taste and nutritional value of this kind of milk, which however needs further investigation.

### 6.2. High-Pressure (HP) Processing

HP processing is a non-thermal processing method that can replace traditional thermal processing and is widely used in the food processing industry. HP treatment is able to kill microorganisms by disrupting the fluidity and integrity of their cell membranes and inactivate specific enzymes by changing their spatial structures under high pressures at room temperature to extend the shelf-life of raw materials and food products in order to ensure food safety while reducing quality loss in the processed products. HP treatment does not lead to thermal degradation of food ingredients, does not impair the organoleptic, nutritional and physicochemical properties of food, does not affect the composition of fatty acids, and minimizes the use of chemical additives [43,44].

HP treatment is also widely used in the pre-treatment of milk. HP treatment requires the use of extremely high pressures (typically between 100 and 1000 Mpa) for a specific period of time [58]. This allows HP treatment to achieve a reduction in the population of microorganisms in milk, such as *E. coli*, without the aid of heating [44].

In addition, the pressure during HP treatment also affects the properties of the milk itself, such as temperature, pH, fat globule size, casein micelle particle size and protein distribution and hydrophobicity [46]. Studies have shown that the diameter of the particle size of untreated goat’s milk increases after 14 days of refrigeration while, in HP-treated goat’s milk, the increase in the above parameters is inhibited or eliminated after higher pressure (>200 MPa) treatment, and even 400 Mpa HP-treated goat’s milk has no significant difference in particle size during storage [43].

In terms of effects on lipids, HP treatment has been shown to alter the size of the fat globules and the composition of the MFGM, with pressure slightly affecting the size of the fat globules and temperature parameters having a greater effect on the size [44]. Processing milk at temperatures above 25 °C produces smaller fat globules, while the effect is opposite at lower temperatures. However, the HP treatment itself has been shown to have little to no effect on the lipid composition of milk. In addition, under mild pressure (250 MPa) conditions without heating, enzymatic lipolysis is facilitated and, due to the release of free fatty acids, as well as the production of mono- and ditriglycerides and additional volatile compounds, leads to undesirable flavors and aromas. Therefore, the effects of high pressure on milk fat and fat globules need to be studied in more depth, especially since these changes are related to the stability of the emulsion system and quality changes in the final products [44].

HP treatment also alters casein micelle size. The effect of HP treatment on the electrostatic and hydrophobic interactions leads to the breakdown of submicelles, followed by the dissociation of casein moieties from micellar casein, after which they re-aggregate and rebind, causing changes in casein micelle size [43]. HP treatment can also cause protein denaturation, mainly by causing the unfolding and destruction of different bonds and interactions, which changes the native structure of proteins. These changes in milk proteins also lead to alterations in their functions and properties. During HP treatment, the size, composition and hydration of casein micelles undergo structural changes. However, the specific variation and the extent of these changes depend on the processing conditions. During HP treatment, the water is compressed, which causes the hydrophobic bonds of the casein micellar components to be broken and then changes the light transmission of the milk, and the minerals are also dissolved (mainly micellar phosphate). A study indicated that HP treatment exacerbated the change in casein micelle size [44]. HP treatment after the application of 500 MP severely affected and changed the size and structure of casein micelles (the spherical structure became irregular, the structural integrity was lost, and the size decreased), and these changes were thought to be responsible for additional effects on the properties of milk, such as changes in color and stability, rheological properties, and pH [44].

HP treatment also has an effect on whey protein. The whey protein in HP-treated milk undergoes reversible unfolding due to pressure, which allows the water molecules in the medium to penetrate the hydrophobic region of molecules, leading to conformational change of the protein and eventually the formation of aggregates. This structural change promotes the improvement of some specific functions of the milk, such as hydrophobicity, solubility, gelatinity, firmness and emulsifying properties, which can improve the quality of other dairy products, such as cheese and yogurt. However, the specific variation and the extent of these changes depend on the processing conditions. For instance, due to the interaction between denatured *β*-lactoglobulin and *κ*-casein, a lighter pressure of about 250 MPa may increase the size of the micelles; however, these changes are reversible [44]. Temperature and pH also affect the change in micelle size during processing, and it was found that, as the pH increased, the temperature increased the micelle size [44]. At the same time, HP treatment has a dual effect on enzymes, which can be activated or inhibited depending on the intensity of the pressure, the type of enzyme, and the temperature. Pressures below 350 MPa can increase the activity of the enzyme because the partial unfolding of the enzyme and protein-based substrate is conformationally flexible, thus facilitating the interaction between them. Inactivation is known to begin at pressures above 400 MPa, and inactivation may increase as pressure increases. However, the degree of inactivation is affected not only by pressure level, but also by processing time, enzyme type, milk composition, and pH level [44].

During the processing of milk, the degradation of lipids, proteins, and other milk components leads to changes in the flavor profile of final products, which is associated with the formation of volatile compounds, such as aldehydes and sulfur compounds [12]. Overall, however, HP treatment offers an alternative to heat preservation, pasteurization, and sterilization in dairy technology, and allows for the development of new products with the desired texture, flavor, and functional properties [43].

### 6.3. Homogenization

In the food industry, homogenization usually refers to the more even dispersion of fat, protein and other components in food through physical methods (such as high-pressure homogenizers), so as to improve the taste, texture and stability of food. Homogenization is widely used in the dairy industry to prevent creaming of milk during storage [34]. For example, in milk processing, homogenization can cause the fat globules to break into smaller particles, preventing the fat from floating and making the milk more delicate. Homogenization is also used to produce reformulated milk with skimmed milk powder and milk fat, as well as to produce filled milk with non-dairy lipids, such as vegetable oils [1].

#### 6.3.1. Effect of Homogenization on Milk with Original or Restructured Single Emulsion Structures

Homogenization can inhibit emulsion creaming during storage of liquid dairy products by reducing the size of milk fat globules [45]. The main mechanism is that the milk is forced through a narrow gap under high pressure in the high-pressure homogenizer, creating a strong shear force, impact force, and cavitation effect, which work together to break the fat globule. Homogenization also prevents milk fat globules from clumping together by converting them into smaller particles. Thus, homogenization is able to promote fat dispersion [33]. For example, homogenization can reduce the size of milk fat globules, thereby improving milk stability [59]. Studies have also shown that homogenization can reduce the size of goat milk fat globules, thereby increasing the interaction between fat globules and casein, which is beneficial to the stability of the emulsion structure of goat milk [60]. Turbulence, shear, and cavitation during homogenization are the main forces that lead to fat globule rupture [11,12]. However, the extent of reduced size of fat droplets by homogenization (5–25 MPa) highly depends on the pressure. Sometimes, fat globule aggregation may occur during homogenization, but this can be eliminated by two-stage homogenization [56].

Studies have shown that homogenization reduced the size of milk fat globules in milk, increased their number and surface area, and altered MFGM properties [12,13,15,56]. As a result, the milk fat globules are not completely covered by the MFGMs. Therefore, the surface-active components in the aqueous phase of milk emulsion, such as casein and whey protein, spontaneously adsorb to the surface of the fat globules, form new interfaces, and provide stability against coalescence [1,2,56]. However, at the time of homogenization to reduce the size of milk fat globules, proteins can be partially replaced by phospholipids, which is determined by the polarity of phospholipids and their concentration on the outer bilayer of MFGMs [34].

The above-mentioned changes in emulsion structure caused by homogenization may affect the digestibility of milk. The reduced size of the milk fat globules may result in a faster digestion rate. However, homogenization of natural milk fat globules does not appear to improve the lipolysis rate, as the effect of increased surface area may be offset by the presence of proteins at the interface of lipid droplets, and homogenized droplets tend to aggregate more quickly than natural milk fat globules under gastric conditions [11,13,56]. Studies have shown that homogenization does not appear to have a significant effect on the final degree of milk fat digestion, but following thermal treatment appears to reduce the overall degree of lipid digestion of homogenized samples [2]. The probable reason was that heating above the thermal denaturation temperature of the adsorbed whey protein can promote the unfolding and interfacial cross-linking of the whey protein. As a result, the interfacial structure and fat globule aggregation are significantly changed, which reduces the speed and degree of fat globule digestion [2]. However, higher temperatures during homogenization can improve the efficiency of homogenization [12], but previous studies showed that milk fat globules (MFGs) after homogenization and thermal treatment had higher flocculation rates than natural MFGs [36].

In addition, different homogenization methods (e.g., shear homogenization, atmospheric homogenization, high-pressure homogenization (HPH), and ultra-high-pressure homogenization (UHPH)) may have different effects on the structural changes of the milk described above.

The application of HPH can increase the temperature of milk [15,34]. As a result, milk can be homogenized at a lower initial temperature compared to atmospheric pressure homogenization, which eliminates the heating phase and simplifies the process [34]. HPH is more effective in generating small emulsion droplets [28]. This technique can replace atmospheric homogenization or pasteurization with a lower pasteurization temperature. At the same time, it effectively eliminates pathogenic microorganisms and improves the quality of milk emulsions [15,34].

HPH treatment is also able to affect the adsorption of proteins on the surface of fat globules, lead to the denaturation of whey proteins, and alter the structural properties of casein micelles, thereby altering the functional properties of proteins and their condensation [45]. It was found that the adsorption of milk proteins on the surface of milk fat globules was promoted by the application of HPH in the pressure range of 100–200 MPa and the temperature range of 20–40 °C, and the adsorption capacity increased with the increase of temperature [34]. The properties of these new interfaces (i.e., stability and viscosity) can also be significantly altered during thermal treatment, due to changes in the conformation and interactions (i.e., protein–protein and protein–fat interactions) of the different types of proteins present (e.g., casein and whey protein) [12]. In the temperature range of 20 °C to 40 °C, the molecular movement of milk protein intensified with the increase in temperature [12], which was conducive to its adsorption and rearrangement on the surface of milk fat globules. In addition, elevated temperatures may also promote protein–protein and protein–fat interactions, further enhancing the stability of the emulsion. However, excessively high temperatures can also cause protein denaturation or aggregation, which is detrimental to the stability of the emulsion.

UHPH is a homogenization method based on the same principles as atmospheric pressure homogenization, but working at higher pressures. UHPH can operate at pressures as high as 400 Mpa, compared to 20–50 MPa homogenized at atmospheric pressure [38,54]. Cavitation, friction, turbulence, and shear stresses are the forces that arise during the UHPH process. UHPH is also able to reduce milk microbial counts, denature whey protein, inactivate intrinsic lactases, such as plasmin and alkaline phosphatase, and drastically reduce fat globule size. Studies have shown that UHPH could reduce the size of fat globules (down to a fraction or even less) and enhance the activity of natural lactoprotein lipase [54]. However, the study also showed that high-temperature short-time (HTST) thermal treatment (72 °C, 15 s) was able to completely inactivate natural lipases that cause liquid lipolysis, such as natural lactoprotein lipase [54].

Shear homogenization refers to crushing and refining materials through extrusion, impact and shear, so that the dispersed phase is distributed in the immiscible continuous phase to achieve the purpose of uniform mixing. Studies have shown that shear homogenization of buffalo milk improved its texture and made its gel firmer with a higher storage modulus, a smaller hysteresis area, and better deformation recovery ability. In addition, the newly formed MFGMs after homogenization are different from the original MFGMs, and the former are mainly composed of membrane fragments and casein, in which a newly formed dense protein layer is covered by the MFGMs. These newly formed MFGMs are cross-linked with the surrounding milk protein matrix, resulting in higher gel strength. However, homogenization also has side effects, with studies showing that shear homogenization increased the total content of free fatty acids in buffalo milk compared to untreated buffalo milk, which may lead to fat-soluble rancidity [36]. Compared with raw milk, shear homogenization could increase free saturated fatty acids by 2.75~3 g per 100 g of fat [36].

#### 6.3.2. Effect of Homogenization on Milk with Mixed Emulsion Structure

In the study of milk with mixed emulsion structure, after homogenization under atmospheric pressure, milk is rich in substances that can accumulate on the surface of newly formed fat globules, accompanied by a reduction in the size of fat globules. HPH can also reduce the diameter of milk fat globules, increase the surface area of milk fat globules, and promote the emulsification of excess fat in dairy products. Both conventional homogenization and HPH can improve the thermal stability (HS) of milk emulsions, while HPH can increase the viscosity of emulsions [15]. In a study on the effect of homogenization conditions on the oxidative stability of fish oil-fortified milk, it was found that homogenization conditions (e.g., temperature and pressure) had no effect on the oxidative stability of milk emulsions containing stable fish oil, and that the actual composition of the oil–water interface (e.g., protein composition) was more important than the total surface area itself [5].

#### 6.3.3. Effect of Homogenization on Milk with Double Emulsion Structure

In the study of the formation of a double W_1_/O/W_2_ emulsion in skim milk using minimal food-grade emulsifiers (i.e., skim milk in the inner and outer aqueous phases and sunflower oil in the oil phase), ultrasonic waves were used in the first emulsification step and different high-shear techniques (i.e., ultrasonic and HPH) were used in the second emulsification step [28]. Results showed that an increase in HPH pressure or an increase in the duration of ultrasound resulted in a smaller droplet diameter, but HPH was more efficient at producing small emulsion droplets because the mechanism of HPH is not to produce droplets larger than the valve gap. However, for a given energy load, either ultrasound or HPH could form a double emulsion with similar size and similar degree of encapsulation [28]. Therefore, it can be concluded that homogenization can reduce the size of the fat globules in milk with a double emulsion structure and, at the same time, increase the surface area of the milk fat globules.

### 6.4. Ultrasonic Treatment

Ultrasound treatment is a promising technology for disrupting particles and can be applied to food processing [4,61]. Ultrasound refers to sound waves with frequencies above 20 kHz that are imperceptible to the human ear and are divided into two categories: power ultrasound and diagnostic ultrasound. Diagnostic ultrasound ranges from 2 MHz to about 15 MHz and is widely used in the medical field. In contrary, power ultrasound, which ranges from 20 kHz to about 1 MHz, has been applied in food processing [4]. For example, powder ultrasound has been used to form stable functional skim milk with the bioactive substance (i.e., black seed oil) [10].

#### 6.4.1. Effect of Ultrasonic Treatment on Milk with Original or Restructured Single Emulsion Structures

Ultrasonic treatment is considered to be an effective processing method, because it can lead to acoustic cavitation in liquids. When energy passes through a liquid medium, it causes the formation, growth, and rupture of bubbles, resulting in strong local shear forces and turbulence with an increase in temperature. These stresses and repeated disintegration of bubbles can disrupt the MFGMs, creating spatial and transient stresses on the surface of the fat globules and particles, which can lead to particle breakage. Many studies have proved that ultrasonic waves mechanically create air bubbles in fluids through cavitation, resulting in a reduction in particle size [4,28,33,36,62].

For milk, the turbulence associated with acoustic cavitation enhances the partial mobility of milk fat globule particles, thereby improving the aggregation between co-aggregated protein and the newly formed MFG-protein complex. As a result, milk gels produced from ultrasonicated milk showed improved gel properties, such as gel strength, elasticity and hardness, viscosity, and water holding capacity, while reducing gelling time [36]. However, prolonged ultrasonic treatment has been reported to have adverse effects on milk gel because whey proteins dissociate from micellar aggregates and MFG flocculates to form homogeneous clusters, resulting in a weak gel network with high synergistic effects [36].

During ultrasonic treatment, the shear forces generated by acoustic cavitation can also cause changes in the protein particles in the milk emulsion. The strong cavitation force usually leads to partial cleavage of the hydrophobic interaction between whey protein molecules, resulting in reversible or irreversible denaturation, depending on the ultrasound intensity [36]. It has been found that whey proteins and whey–whey aggregates in milk were denatured and soluble whey–whey/whey–casein aggregates were formed, which further interacted with casein micelles to form micellar aggregates during the first 30 min ultrasound treatment; however, prolonged treatment with ultrasound results in the destruction of some of the whey protein from these aggregates [4]. Partially denatured whey protein can provide stability to emulsified droplets, allowing for the production of emulsion droplets with smaller sizes [63]. In addition, the physical forces of acoustic cavitation have no effect on the integrity of the casein micelles but result in a slight reduction in their size. However, studies have shown that small changes to the protein by ultrasound do not alter the viscosity of milk [4].

Ultrasonic treatment can also be used to assist in producing milk with a restructured single emulsion structure. Studies have shown that ultrasound-assisted emulsification can be used to prepare stable turmeric oil-loaded milk emulsions [64]. Ultrasound-assisted emulsification has considerable advantages over conventional methods, such as obtaining submicron-sized milk fat globule particles, narrow particle size distributions and more stable emulsions, and low energy requirements, and can be used as a simple and low-cost processing technique [16,64]. Ultrasound-assisted emulsification is a two-step process in which Rayleigh–Taylor instability drives the formation of droplets of the dispersed phase in a continuous phase, followed by resulting droplets in the second step, which are broken into tiny droplets by a shock wave generated by instantaneous cavitation. The formation and final size of the droplets are influenced by a number of operational factors, including the presence of surfactants. The main role of surfactants is to reduce the interfacial tension so that less energy is required to produce the droplets, and also to adsorb on the surface of the newly prepared droplets to prevent droplet recoalescence, resulting in a stable emulsion [64].

Ultrasonic treatment can also be used in combination with homogenization to process milk. The main purpose of ultrasonic homogenization is to reduce the size of fat globules, but also to increase the stability and consistency of the resulting milk emulsion, and to reduce the separation of fat and aqueous phases during consumption and storage. By controlling the temperature and shortening the homogenization duration, the negative effects of ultrasonic treatment, such as lipid oxidation, can be avoided [46].

#### 6.4.2. Effect of Ultrasonic Treatment on Milk with Double Emulsion Structure

The physical and chemical effects produced by cavitation bubbles have many practical applications in milk with a double emulsion structure. In the study of the preparation of W_1_/O/W_2_ emulsions containing skim milk and sunflower seed oil with low-frequency ultrasonic treatment, sonication was used in the preparation of both W_1_/O and W_1_/O/W_2_ emulsions, and the results showed that the resulted double emulsion droplets were relatively stable within 7 days [16]. The results of above study suggested that, for a given energy load, either ultrasound or HP treatment can form a similar size and similar degree of encapsulation of milk with a double emulsion structure [28]. As expected, an increase in the duration of ultrasound or HP treatment pressure could result in a smaller droplet diameter. However, increasing the ultrasonic power may lead to greater shear forces, which results in more emulsion droplets being destroyed and then reduces the retention of internalized aqueous droplets [16]. Compared with HP homogenization, ultrasonic treatment has an inherent randomness due to its mechanism of acoustic cavitation, and the reduction in droplet size is random, resulting in a larger size distribution. Therefore, in this case, the presence of larger droplets may lead to a higher encapsulation efficiency at the time of encapsulation, but this may also reduce the stability of the double milk emulsion [28].

Another application example is the encapsulation of glycyrrhizic acid (GA) with an ultrasound-assisted milk-based double emulsion, in which ultrasonic treatment was used in the preparation of both W_1_/O and W_1_/O/W_2_ emulsions [17]. Studies have shown that sonication was able to reduce droplet size; However, prolonged ultrasonic treatment could lead to droplet rupture in the original emulsion, a decrease in pH and an increase in temperature, resulting in uncontrolled release of the encapsulated compound and lower encapsulation efficiency [17]. Therefore, an appropriate sonication time should be selected to control the encapsulation efficiency [16,17,18].

### 6.5. Membrane Emulsification (ME)

ME involves the use of low compression to allow the dispersed phase to permeate through the membrane with a specific pore size under the action of different pressures into the continuous phase. In contrast to homogenization, the resulting droplet size is mainly controlled by the choice of membrane rather than by the generation of turbulent droplet breakage. The technique is very attractive due to its simplicity, potentially lower energy requirements, the need for less surfactant and the resulting narrow droplet size distribution [1,51]. The advantages of ME in the food processing industry may come from its low shear properties and mild processing conditions, especially for the preparation of double emulsions, structural phases with fine droplets, and microcapsules. Therefore, in contrast to traditional emulsification methods (e.g., homogenization), small and monodispersed droplets can be produced without the use of high shear stresses that cause internal droplets to escape, which is more effective in maintaining the microstructures and nutrients in emulsion food products [51]. Another advantage of ME is the amplification capability of the membrane device. By adding more fibers to the unit, increasing the fiber length, and using the unit in parallel, the flow rate can be easily increased to achieve the desired conversion rate and productivity. The limitations of the ME process may be related to the low flux and fouling of monodisperse emulsions. These shortcomings can be addressed by other operations, such as premixed processing, as well as rotating or vibrating membrane devices [51].

ME technology is divided into two types: direct ME and premixed ME: (i) in direct ME, a continuous phase flows tangentially to the membrane surface, while the dispersed phase is squeezed through the pores of the membrane, and the droplets of the dispersed phase grow at the openings of the pores in the membrane and separate when they reach a certain size; (ii) premixed ME is based on the formation of a coarse emulsion by traditional mechanical techniques and then passing it through a membrane to obtain a narrow droplet size distribution [1,65].

In ME, different types of membranes can be used, such as Shirasu-porous glass (SPG) membranes, ceramic membranes, and silicon and silicon nitride membranes [1,51]. In the food industry, different membranes have their own specific application scenarios. Inorganic membranes (e.g., ceramic and SPG membranes) are suitable for the preparation of food emulsions that require strict hygiene, high-temperature sterilization or chemical resistance. Silicone-based membranes may be more suitable for some food emulsions that require precise control of droplet size. ME is a suitable technology for emulsified oils and milk. It has been reported that milk proteins could stabilize emulsions without the use of any external reagents as surfactants, achieve encapsulation at an oil concentration of 30 wt%, and obtain droplet sizes that are approximately 2 to 6 times the size of the membrane pore [1].

In the study of the preparation of O/W emulsion containing fish oil by premixed ME method, the study showed that the emulsion produced by premixed ME has a smaller droplet size distribution and is more monodisperse than the emulsion produced by the rotor-stator [53]. Another study indicated that ME could be successfully used to produce astaxanthin-containing O/W emulsions with significantly narrow droplet size distribution, and that the average size of the droplets could be adjusted by varying experimental parameters, such as pressure and dispersed phase fraction, and small mean diameter values were obtained at high pressure and low disperse phase fractions [65].

At present, there are no studies on the effect of membrane emulsification on the structure of mixed milk emulsions. However, the predicted effect of membrane emulsification on the structure of the mixed milk emulsion is mainly in terms of droplet size, which can produce relatively small droplets and may have a higher encapsulation efficiency. In future, understanding the effect of different material-based membranes with different pore sizes on mixed milk emulsions can help to provide a basis for selecting the right membrane. The experimental parameters in the membrane emulsification process, such as pressure, temperature, flow rate, emulsifier type and concentration, need to be investigated for regulating the structure and properties of mixed milk emulsions. In terms of milk with a double emulsion structure (i.e., W_1_/O/W_2_), primary emulsions can be prepared by conventional methods or by ME. The mild conditions of ME are particularly useful for the second emulsion step, which prevents the rupture of the double emulsion droplets [51].

### 6.6. Microfluidization

Microfluidization is an emerging technique and the most effective method for generating uniformly distributed droplets [35,66]. Due to its high cost and production limitations, its applicability on a larger scale is limited, but microfluidization has an important role in various studies due to its advantages such as short processing time, low thermal effect, and almost no nutrient loss. Therefore, microfluidization technology can produce emulsified products with uniform particle size, good nutrient retention and high stability, which meets the production requirements of high value-added products. In such cases, the advantages of microfluidics may indeed outweigh its high costs. Microfluidization is not only used in the preparation and production of emulsions, nanoparticles, and beverages, but is also used to process some food materials to improve their specific properties and thus is suitable for new applications [67]. In principle, the production of emulsions using microfluidizers is the most energy-efficient and suitable for high-throughput processing, though the cost of maintenance is high [16].

The formation of milk emulsion using a microfluidizer usually involves two steps. First, a high-shear agitator is used to mix the oil, emulsifier, and water phase together to form a coarse emulsion. Secondly, pneumatic pressure is used to force the coarse emulsion through the microfluidizer. After the coarse emulsion enters the microfluidizer, it is split into two separate streams, causing them to collide with each other at high speed. This process generates strong destructive forces, such as cavitation, turbulence, and shear, which effectively disrupt the large droplets in the coarse emulsion, resulting in the formation of very tiny droplets [66,67,68,69,70,71]. In addition, the frequent collisions occurring between the particles (e.g., fat globules and protein aggregates) and between the particles and the microchannel walls are also able to break up larger particles and reduce their size, thus improving the uniformity and stability of milk. Crude emulsions prepared by high-shear mixers can reach a particle size of 1.5 μm, while emulsions prepared by microfluidization processes have a smaller particle size (≥200 nm) [68]. The results of a study in the production of emulsion containing fish oil showed that microfluidization could form stable nano=emulsions, and that, when the average droplet size of nano=emulsions was around 200 nm, it could be used to improve their absorption in the digestive tract [68]. Another study showed that microfluidization techniques allowed emulsions to have droplet sizes in the nanometer range (<500 nm) with a single-modal particle size distribution and good stability [70].

In general, the size of the emulsion decreases with increasing pressure and duration within the optimal microfluidization pressure range. However, after exceeding the optimal treatment conditions, the emulsion size may increase or barely change, because the emulsion droplets break and aggregate [69,72]. A study indicated that that, during the process of emulsification homogenization, the shear, turbulence and collision caused by microfluidization could simultaneously change the size of oil droplets in an O/W emulsion stabilized by natural pea globulin and the structure of the pea globulin-based interfacial membrane [73]. In addition, when the microfluidization pressure increased, the emulsion stability of the liquid emulsion had two opposite effects, either an improvement in the emulsion stability or a decrease in the emulsion stability of the liquid emulsion, which might be a consequence of flocculation [73]. Further research should be carried out to determine the specific effects of different pressure gradients, temperatures, and processing flow on the droplet size in milk. However, it has been indicated that, when the microfluidization pressure was held constant, the particle sizes increased as the milk fat concentration was increased, indicating that higher microfluidization pressure might be needed for milk containing higher levels of fat to obtain stable final products [74].

In terms of the effect of microfluidization on the digestive behavior of milk, it has been indicated that microfluidization treatment could promote in vitro and in vivo protein digestibility of milk [75]. This may be due to the breakage of fat, as well as protein molecules, into a very small size with increased surface area as a result of microfluidization, thereby interacting more fully with digestive enzymes and accelerating the release of nutrients, such as fatty acids and amino acids. However, more research is needed to further clarify this relationship and provide a basis for the development of more efficient nutrient delivery systems.

### 6.7. Freezing

Freezing is a common technique in the food industry to preserve food and extend its shelf-life. Food is exposed to extremely low temperatures, usually below freezing, which slows down bacterial growth and chemical changes, thus preserving the freshness and quality of food. Freezing can also be applied to the storage of milk, and freezing has a certain effect on the original emulsion structure in milk. When milk is frozen, several reactions occur, which are related to the freezing temperature or freezing time. There are two unstable phenomena in milk when it is frozen. One is fat separation, which occurs during the cooling process of milk in the early stage of freezing. The other is protein flocculation, which depends on the final storage temperature and the time of frozen storage [48].

When milk is stored at low temperatures, the fat globules may undergo partial coalescence. The possible mechanism is that freezing can lead to the crystallization of triglyceride nuclei, and the fat crystals can protrude from the surface of the fat globules and pierce the neighboring MFGMs, partially coalescing to produce large, irregularly shaped particles or a continuous network of aggregated fats, which tend to rapidly form fat creaming. The partially coalesced fat globules themselves can further coalesce as the temperature rises and the fat crystals melt. During the partial coalescence, some components in MFGMs can be released into the water phase [11,56,60].

In addition to the changes in fat, freezing also has an effect on the non-fat compositions in milk, mainly the balance of salt. This can be explained by the partial freezing of water and the formation of a supersaturated salt solution, increasing the ionic strength and thus the osmotic pressure of the system [60].

## 7. Future Outlook

The future development of milk with different emulsion structures may focus on the following areas:

(i) The design of functional milk: Future research may focus on the design of multifunctional milk, which can give milk more functionality by changing the surface structures of milk fat globules, remodeling its emulsion structures, and introducing other functional components into milk. Moreover, in the relevant practical applications, it is necessary to select the appropriate additives (e.g., oil from different sources with various properties) and processing processes according to the specific production needs and product characteristics to achieve the best effects, which also needs more in-depth and systematic research.
(ii) The innovation in emulsifying technology: The innovation of emulsification technology helps to improve the stability and texture of milk with remodeled emulsion structures. On the one hand, although some emulsifying technologies (e.g., homogenization and ultrasonic treatment) have been used for preparing milk with original, restructured single, mixed, or double emulsion structures, some novel emulsifying technologies (e.g., ME and microfluidization) are still limited to remodeling the original emulsion structures in milk. These novel emulsifying technologies may show good performance to improve the properties of milk, which however needs further investigation for confirmation. On the other hand, the combination of different emulsifying technologies (e.g., the combination of homogenization and ultrasonic treatment) may show synergetic effects. Therefore, more diverse combinations of different emulsifying technologies with different working mechanisms to overcome their respective shortcomings in the production of milk with different emulsion structures may also be an interesting research topic in future.
(iii) The development of gelled dairy products with different emulsion structures and functions: Previous studies mainly focus on the fabrication of emulsion structures in milk for various purposes, but research on the processing properties of milk with remodeled emulsion structures and the functional properties of the relevant processed products are still insufficient. Therefore, more efforts are needed to understand how remodeling emulsion structures in milk affects its subsequent processing performance (e.g., fermentation, concentration, and curdling) and the sensory and functional properties of the resultant final dairy products (e.g., yogurt and cheese).

## 8. Conclusions

Milk with its original emulsion structure has problems, such as short shelf-life and high number of microorganisms, which limit its economic value. The treatment of milk using different processing technologies, including thermal treatment, high-pressure treatment, homogenization, sonication, micro-fluidization, freezing and membrane emulsification, can improve the stability of milk and reduce microbial counts, thereby extending its shelf-life. Among them, heat treatment and UHP technologies have been most widely used in reducing the number of microorganisms in milk, due to their advantages in low cost and good maturity. In addition, these processing methods can have an impact on the microstructure of milk, such as altering the structural and functional properties of milk proteins, the size and surface area of milk fat globules, and the structural and digestive properties of milk lipids. In addition, in order to improve the taste and texture of milk (especially skim milk) and improve its nutritional value and functionality, the original emulsion structure of milk can be remolded by using cod liver oil, flaxseed oil and sunflower oil to prepare final products containing restructured single, mixed or double emulsion structures. Similarly, different processing methods can also affect the microstructure of liquid emulsions containing the three remodeled emulsion structures above, but current research mainly focuses on homogenization and sonication. In addition, changes of the structural properties of milk during a single processing method have been widely investigated in previous studies, while the effect of multiple processing methods on milk is rarely reported. In summary, the structural remodeling of milk can improve the texture, taste, stability and nutritional value of dairy products. Further research on remodeling the emulsion structure of milk will promote the application and development of milk emulsion, and provide more innovative and improved directions for the food industry.

## Figures and Tables

**Table 1:** Examples of milk containing mixed emulsion structures.

The Type of Admixture	Original Milk	Added Oil Phase	References
O/W emulsion	Whole milk	8–12 wt% flaxseed oil emulsion (addition amount of 5 wt%)	[20]
	Whole milk or milk with 2% fat	25 wt% algal oil emulsion (final concentration with 5 wt% algal oil added)	[21]
Oil	Whole milk	Flaxseed oil (addition amount of 10 wt%)	[7]
	Whole milk	Cod liver oil (addition amount of 5 wt%)	[6]
	Whole milk	Ghee and canola oil blends (addition amount of 20–50 wt%)	[8]
	Milk with 1.5% or 3.5% fat	Cod liver oil (addition amount of 0.5 wt%)	[22]
	Milk with 3.5% fat	Buttermilk powder (addition amount of 0.3–1.5%, *w*/*v*)	[15]
	Milk with 4.5% fat	Chia oil and α-lipoic acid nanoliposomes (addition amount of 20 wt%)	[23]
	Milk with 0.5 wt% and 1.5 wt % fat (1:1)	Cod liver oil (addition amount of 0.5 wt%)	[5]
	Milk with 0.5 wt% and 1.5 wt % fat (1:1)	Cod liver oil (addition amount of 0.5 wt%)	[24]
	Remix concentrated milk	Phospholipids (addition amount of 0.0–0.2 wt%)	[25]
	Compound evaporated milk	Cream residuum powder and sweet buttermilk powder (addition amount of 0–6 wt%)	[26]
	Full-fat donkey milk	Sunflower oil (addition amount of 1.6%, *v*/*v*)	[9]

*w*/*v* mass solubility, *v*/*v* volume ratio, and wt% mass percentage.

**Table 2:** Examples of milk containing a double emulsion structure.

Inner Aqueous Phase W_1_	Aliphatic Phase O	Outer Aqueous Phase W_2_	Emulsifiers	References
Distilled water (20 g)	(LT, BF, HS, or SO, 80 g)	Reconstituted milk (80 g, containing 10.0 wt% skimmed milk powder)	PGPR	[30]
Distilled water (20 wt%)	Olive oil (71.9 wt%)	Aqueous solution of biopolymers (80 wt%)	WE, OE, and GG	[31]
Ferrous sulfate (1%, *w*/*v*) in distilled water (40 wt% to W_1_/O emulsion)	MCT (60 wt%)	30% WPI solution (75 wt% to W_1_/O/W_2_)	PSML, and PGPR	[32]
0.001 M phosphate buffered saline containing 0.2%, *w*/*v* vitamin B_12_ (10–30% oil-based)	Sunflower Oil (70–90%)	Skim milk (75–95%)	PGPR	[17]
0.001 M phosphate buffered saline containing 0.2%, *w*/*v* vitamin B_12_ (10–30% oil-based)	Sunflower Oil (70–90%)	Skim milk (75–95%)	PGPR	[18]
Skimmed milk (30 wt%)	Sunflower Oil (70 wt%)	Skim milk (95 wt%)	PGPR and lecithin mixture	[28]
Skimmed milk (10 wt% oil-based)	Sunflower Oil (90 wt%)	Skim milk (80–95 wt%)	Span 80	[16]
Skim milk (40 wt%)	Canola oil or anhydrous milk fat (60 wt%)	Skim milk (80 wt%)	Sunflower lecithin, and PGPR	[27]

[Abbreviation: low trans vegetable fat (LTVF), refined bovine fat (BF), partially hydrogenated soybean oil (HS), refined sunflower oil (SO), hydrophilic emulsifier (WE), hydrophobic emulsifier (OE), gellan gum (GG), medium-chain triglyceride (MCT), whey protein isolate (WPI), poly-oxyethylene sorbitan monolaurate (PSML), polyglycerol poly-ricinoleate (PGPR), Span 80: lipophilic surfactant], [*w*/*v* mass solubility, *v*/*v* volume ratio, wt% mass percentage].
